# Heterogeneity in colorectal cancer incidence among people recommended 3-yearly surveillance post-polypectomy: a validation study

**DOI:** 10.1055/a-1217-0155

**Published:** 2020-08-19

**Authors:** Emma C. Robbins, Kate Wooldrage, Iain Stenson, Kevin Pack, Stephen Duffy, David Weller, Theodore Levin, Carol Conell, Suzanne Wright, Claire Nickerson, Jessica Martin, Amanda J. Cross

**Affiliations:** 1 Cancer Screening and Prevention Research Group (CSPRG), Department of Surgery and Cancer, Imperial College London, United Kingdom; 2 Centre for Cancer Prevention, Wolfson Institute of Preventive Medicine, Queen Mary University, London, United Kingdom; 3 Centre for Population Health Sciences, University of Edinburgh, Edinburgh, United Kingdom; 4 Division of Research, Kaiser Permanente Northern California, Oakland, California, United States; 5 Public Health England (PHE) Screening, Sheffield, United Kingdom

## Abstract

**Background** Colonoscopy surveillance is recommended for patients at increased risk of colorectal cancer (CRC) following adenoma removal. Low-, intermediate-, and high-risk groups are defined by baseline adenoma characteristics. We previously examined intermediate-risk patients from hospital data and identified a higher-risk subgroup who benefited from surveillance and a lower-risk subgroup who may not require surveillance. This study explored whether these findings apply in individuals undergoing CRC screening.

**Methods** This retrospective study used data from the UK Flexible Sigmoidoscopy Screening Trial (UKFSST), English CRC screening pilot (ECP), and US Kaiser Permanente CRC prevention program (KPCP). Screening participants (50 – 74 years) classified as intermediate-risk at baseline colonoscopy were included. CRC data were available through 2006 (KPCP) or 2014 (UKFSST, ECP). Lower- and higher-risk subgroups were defined using our previously identified baseline risk factors: higher-risk participants had incomplete colonoscopies, poor bowel preparation, adenomas ≥ 20 mm or with high-grade dysplasia, or proximal polyps. We compared CRC incidence in these subgroups and in the presence vs. absence of surveillance using Cox regression.

**Results** Of 2291 intermediate-risk participants, 45 % were classified as higher risk. Median follow-up was 11.8 years. CRC incidence was higher in the higher-risk than lower-risk subgroup (hazard ratio [HR] 2.08, 95 % confidence interval [CI] 1.07 – 4.06). Surveillance reduced CRC incidence in higher-risk participants (HR 0.35, 95 %CI 0.14 – 0.86) but not statistically significantly so in lower-risk participants (HR 0.41, 95 %CI 0.12 – 1.38).

**Conclusion** As previously demonstrated for hospital patients, screening participants classified as intermediate risk comprised two risk subgroups. Surveillance clearly benefited the higher-risk subgroup.

## Introduction

Adenoma removal reduces colorectal cancer (CRC) incidence and mortality 1 2 3 4 . However, some patients remain at increased risk of CRC following adenoma removal and are recommended surveillance colonoscopy 5 6 7 8 9 . In the 2002 UK surveillance guidelines, patients with adenomas are classified as low, intermediate, or high risk according to risk of subsequent advanced neoplasia 9 . Evidence for the risk group definitions mostly came from studies examining detection rates of advanced adenomas at follow-up 10 11 12 13 , owing to a lack of data on long-term CRC incidence. Evidence to inform the surveillance recommendations was also limited. For example, the recommendation for 3-yearly surveillance in intermediate-risk patients was based on one trial, which reported similar detection rates of advanced neoplasia among patients attending surveillance at 1 and 3 years, and at 3 years only 13 .

Given the lack of high-quality evidence supporting the guidelines, we developed the Intermediate Adenoma (IA) study to examine the appropriateness of 3-yearly surveillance in intermediate-risk patients 14 15 . Through analysis of endoscopy and pathology data from 17 UK hospitals, we found that patients classified as intermediate risk at baseline colonoscopy comprised two risk subgroups. Higher-risk patients were those with an incomplete colonoscopy, colonoscopy of unknown completeness, poor bowel preparation, adenoma ≥ 20 mm or with high-grade dysplasia, or proximal polyps at baseline. In this subgroup, surveillance was highly effective. Among patients without these characteristics, the CRC incidence rate was lower than in the general population without surveillance, indicating that surveillance may not have been warranted.

The IA study was the first to identify heterogeneity in CRC incidence among individuals classified as intermediate risk. We wanted to explore whether these findings apply not only in hospital patients but also in individuals undergoing CRC screening. We therefore conducted a validation study by analyzing data from populations undergoing routine CRC screening. We examined CRC incidence rates and effects of surveillance on incidence rates among screening participants classified as intermediate risk at baseline colonoscopy.

## Methods

### Study design and participants

We created a retrospective study by pooling data from three screening cohorts: the UK Flexible Sigmoidoscopy Screening Trial (UKFSST) 4 16 17 , the English CRC screening pilot (ECP) 18 19 , and the US Kaiser Permanente CRC prevention program (KPCP) 20 . These cohorts were investigated in our previous analyses 15 ; however, for the present study, we obtained updated information on the participants, which provided longer-term follow-up data.

In the UKFSST, 170 432 individuals aged 55 – 64 years were randomized between October 1996 and March 1999 to either once-only flexible sigmoidoscopy screening or usual care, which at the time constituted no CRC screening 4 16 17 . In total, 40 674 participants underwent flexible sigmoidoscopy screening at one of 14 UK hospitals. Colonoscopy was offered to 2131 (5 %) screened participants found to have an adenoma ≥ 10 mm, with high-grade dysplasia, villous or tubulovillous histology, ≥ 3 adenomas, ≥ 20 hyperplastic polyps above the rectum, or malignancy. Participants found to have an adenoma ≥ 10 mm, with high-grade dysplasia, tubulovillous or villous histology, or ≥ 3 adenomas at baseline colonoscopy were offered at least two 3-yearly surveillance colonoscopies. We obtained data on surveillance colonoscopies through 2012, and data on CRC diagnoses and deaths through 2014.

The ECP was part of the UK CRC screening pilot, which was also conducted in Scotland 18 19 . We omitted the Scottish dataset from this study as endoscopy and pathology data were not linked. We collected data from three hospitals in Warwickshire involved in the ECP, for individuals enrolled in the first round of the pilot (September 2000 – June 2002). A total of 185 267 individuals (residents of the pilot areas aged 50 – 69 years) were offered a guaiac fecal occult blood test (gFOBT). Uptake was ~60 % (n = 109 609). Colonoscopy was offered to the 1714 participants (2 %) who had a positive FOBT 19 . Prior to widespread adoption of the 2002 UK surveillance guidelines 9 , surveillance of ECP participants initially varied between hospitals. However, almost all participants with an adenoma ≥ 10 mm, with high-grade dysplasia or villous histology, or ≥ 3 adenomas at baseline colonoscopy were offered 3-yearly surveillance colonoscopy 18 . We obtained data on surveillance colonoscopies through 2012, and data on CRC diagnoses and deaths through 2014.

The KPCP was initiated in 1994 20 . From January 1994 – December 1995, 78 034 flexible sigmoidoscopy examinations were performed in individuals aged ≥ 50 years. Colonoscopy was offered to participants with an adenoma ≥ 10 mm, with high-grade dysplasia, villous or tubulovillous histology, > 1 adenoma, or an adenoma of any size/histology together with a family history of CRC. Surveillance colonoscopy was offered to participants with an adenoma ≥ 10 mm, with high-grade dysplasia, villous or tubulovillous histology, or multiple adenomas at baseline colonoscopy. We obtained data on surveillance colonoscopies through 2006 (or the date the participant left the KPCP, if earlier), and data on CRC diagnoses and deaths through 2006.

### Data collection and management

In each dataset, we identified participants who had a baseline colonoscopy following a positive screening test. We obtained linked endoscopy and pathology data for these participants on baseline and surveillance colonoscopies. We divided endoscopic examinations into visits (one or more examinations performed in close succession to fully examine the colon and remove all detected lesions). Summary values for size, histology, location, and shape were assigned to lesions seen at multiple examinations 15 .

We defined examination quality according to the most complete colonoscopy and best bowel preparation achieved during baseline 14 15 . The KPCP dataset did not contain data on examination quality. Considering the high standard of colonoscopies in the USA 21 , we assumed that all KPCP participants had a complete baseline colonoscopy with at least satisfactory bowel preparation.

We classified participants as low, intermediate, or high risk following the 2002 UK surveillance guidelines: participants were low risk if they had one or two adenomas < 10 mm at baseline; intermediate risk if they had three or four adenomas < 10 mm or one or two adenomas with at least one ≥ 10 mm; or high risk if they had five or more adenomas < 10 mm, or three or more adenomas with at least one ≥ 10 mm 9 .

**Fig. 1:**
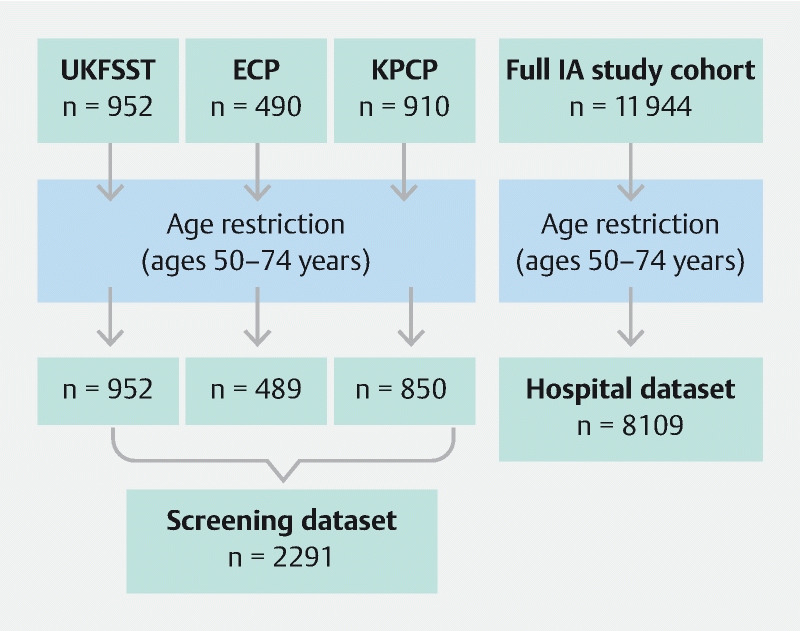
Flow diagram showing creation of the screening and hospital datasets. ECP, English CRC screening pilot; IA, Intermediate Adenoma; KPCP, Kaiser Permanente CRC prevention program; UKFSST, UK Flexible Sigmoidoscopy Screening Trial.

Participants classified as intermediate risk at baseline were included in the present study. We pooled data from the three screening cohorts on intermediate-risk participants to create the “screening dataset.” Although the screening dataset was the focus of our main analysis, we wanted to see how the screening dataset findings compared with those from the IA study 14 15 . As the age range was narrower among screening than IA study participants owing to the age limits of the screening programs, we applied an age restriction to both (50 – 74 years; which captures the recommended age ranges for CRC screening across England, Scotland, and Wales) 22 23 24 . This allowed us to compare the screening dataset with an equivalent subset of the IA study cohort. We called the age-restricted subset of the IA study cohort the “hospital dataset” ( Fig. 1 ).

### Statistical analysis

We examined baseline patient, procedural, and polyp characteristics in the screening dataset and individual screening cohorts. We compared the distribution of baseline characteristics among screening participants with and without surveillance using chi-squared tests.

We calculated CRC incidence rates after baseline including all observation time, allowing for the effect of any surveillance. Time-to-event data were censored at first CRC diagnosis, death, emigration, end of program participation (KPCP), or end of follow-up. Time-at-risk started from the last colonoscopy at baseline.

Using the baseline CRC risk factors identified in the IA study 14 15 , we classified screening participants into lower- and higher-risk subgroups. Participants were classified as higher risk if they had an incomplete colonoscopy, colonoscopy of unknown completeness, poor bowel preparation, adenoma ≥ 20 mm or with high-grade dysplasia, or proximal polyps at baseline. Participants with none of these characteristics were classified as lower risk.

For each risk subgroup, we calculated CRC incidence rates after baseline. We used multivariate Cox proportional hazards models to estimate hazard ratios (HRs) with 95 % confidence intervals (CIs) to compare CRC incidence rates in the subgroups. The models were adjusted for number of surveillance visits, included as a time-varying covariate.

We used univariate Cox proportional hazards models to estimate HRs with 95 %CIs to compare CRC incidence rates in the presence of surveillance (one or more visits) vs. in the absence of surveillance. Exposure to successive surveillance visits started at the last colonoscopy in each visit. Visits at which CRC was diagnosed were not included as surveillance visits because they did not offer protection against CRC.

We conducted Kaplan–Meier analyses to show time to cancer diagnosis and estimate cumulative CRC incidence with 95 %CIs at 10 years. We compared cumulative incidence curves using the log-rank test. For all analyses of CRC incidence, we divided each participant’s follow-up time into two periods; in the absence of surveillance (from start of time-at-risk, censoring at first surveillance) and in the presence of surveillance (from first surveillance, censoring at end of follow-up). We did not stratify the latter period by number of surveillance visits because few CRC cases occurred in each stratum.

We repeated the above analyses for the hospital dataset. We also wanted to see how our data compared with the Bowel Cancer Screening Programme (BCSP) in England and so obtained limited data on a subset of BCSP participants. The subset analyzed included those who underwent colonoscopy from January 2006 to December 2011 following a positive gFOBT, were deemed at intermediate risk, and were referred for surveillance. We obtained data on patient, procedural, and polyp characteristics at baseline colonoscopy and calculated the proportion of participants classified as higher risk. A Data Re-use Agreement permitting use of these data was issued by Public Health England (reference 2015/IA/1).

We performed a sensitivity analysis of the criteria used to classify intermediate-risk individuals into risk subgroups. We first identified baseline characteristics associated with increased CRC incidence rates in the screening dataset when adjustment was made for number of surveillance visits, using multivariate Cox regression. These characteristics were then included in the classification of higher risk, in addition to the IA study risk factors 14 15 .

Analyses were performed in Stata/IC V.13.1 (StataCorp LP, 2013; Stata Statistical Software: Release 13; College Station, Texas, USA). We used a significance level of 0.05 for all analyses.

## Results

There were 952 intermediate-risk participants in the UKFSST, 490 in the ECP, and 910 in the KPCP; of these, 952, 489, and 850, respectively, were aged 50 – 74 years and were included in the screening dataset. The screening dataset therefore comprised 2291 participants. The hospital dataset comprised 8109 patients aged 50 – 74 years, drawn from the IA study cohort of 11944 ( Fig. 1 ) 14 15 .

In the screening dataset, the median age was 61 years (interquartile range [IQR] 57 – 64) and 738 participants (32 %) were female. Approximately 35 % of participants attended one surveillance visit and 44 % attended two or more ( Table 1 ). There was no difference between participants attending surveillance and nonattenders in relation to sex or examination quality; however, attenders were younger and more likely to have had adenomas with tubulovillous histology or high-grade dysplasia at baseline (see **Table 1 s** in the online-only supplementary material).

**Table 1:** Baseline patient, procedural, and polyp characteristics.

	Pooled screening dataset (n = 2291)	KPCP (n = 850)	UKFSST (n = 952)	ECP (n = 489)
No. of surveillance visits, n (%)				
0	499 (21.8)	254 (29.9)	159 (16.7)	86 (17.6)
1	794 (34.7)	376 (44.2)	260 (27.3)	158 (32.3)
≥ 2	998 (43.6)	220 (25.9)	533 (56.0)	245 (50.1)
Sex, n (%)				
Female	738 (32.2)	278 (32.7)	297 (31.2)	163 (33.3)
Male	1553 (67.8)	572 (67.3)	655 (68.8)	326 (66.7)
Age at baseline colonoscopy, years, n (%)				
50 – 54	232 (10.1)	163 (19.2)	0 (0)	69 (14.1)
55 – 59	669 (29.2)	193 (22.7)	381 (40.0)	95 (19.4)
60 – 64	855 (37.3)	196 (23.1)	500 (52.5)	159 (32.5)
65 – 69	410 (17.9)	176 (20.7)	71 (7.5)	163 (33.3)
70 – 74	125 (5.5)	122 (14.4)	0 (0)	3 (0.6)
Year of baseline colonoscopy, n (%)				
1995 – 99	1801 (78.6)	850 (100)	951 (99.9)	0 (0)
2000 – 04	394 (17.2)	0 (0)	1 (0.1)	393 (80.4)
2005 – 10	96 (4.2)	0 (0)	0 (0)	96 (19.6)
Colonoscopy completeness, n (%)				
Complete	2200 (96.0) ^1^	850 (100) ^1^	876 (92.0)	474 (96.9)
Incomplete/unknown	91 (4.0)	0 (0)	76 (8.0)	15 (3.1)
Bowel preparation quality, n (%)				
Excellent/good/satisfactory/unknown	2242 (97.9) 1	850 (100) 1	911 (95.7)	481 (98.4)
Poor	49 (2.1)	0 (0)	41 (4.3)	8 (1.6)
Adenoma size, mm, n (%)				
< 10	265 (11.6)	140 (16.5)	95 (10.0)	30 (6.1)
10 – 19	1573 (68.7)	599 (70.5)	639 (67.1)	335 (68.5)
≥ 20	453 (19.8)	111 (13.1)	218 (22.9)	124 (25.4)
Adenoma histology, n (%)				
Tubular	1109 (48.4)	529 (62.2)	468 (49.2)	112 (22.9)
Tubulovillous	1003 (43.8)	268 (31.5)	396 (41.6)	339 (69.3)
Villous	146 (6.4)	53 (6.2)	63 (6.6)	30 (6.1)
Unknown	33 (1.4)	0 (0)	25 (2.6)	8 (1.6)
Adenoma dysplasia, n (%)				
Low grade	2016 (88.0)	817 (96.1)	811 (85.2)	388 (79.3)
High grade	254 (11.1)	33 (3.9)	121 (12.7)	100 (20.4)
Unknown	21 (0.9)	0 (0)	20 (2.1)	1 (0.2)
Proximal polyps, n (%)				
No	1834 (80.1)	637 (74.9)	817 (85.8)	380 (77.7)
Yes	457 (19.9)	213 (25.1)	135 (14.2)	109 (22.3)

ECP, English CRC screening pilot; KPCP, Kaiser Permanente CRC prevention program; UKFSST, UK Flexible Sigmoidoscopy Screening Trial.

1 Data on examination quality were missing for KPCP participants; we therefore assumed that all KPCP participants had a complete colonoscopy with at least satisfactory bowel preparation at baseline.

During a median follow-up of 11.8 years (IQR 10.3 – 16.1), 37 CRCs were diagnosed among screening participants, giving an incidence rate of 134 per 100 000 person-years (95 %CI 97 – 185). In the individual screening cohorts, the CRC incidence rate per 100 000 person-years was lowest in the KPCP (85, 95 %CI 41 – 179), followed by the UKFSST (149, 95 %CI 97 – 228) and ECP (170, 95 %CI 89 – 327) ( Table 2 ).

**Table 2:** Long-term incidence rates of colorectal cancer after baseline colonoscopy.

Dataset	Examination/screening modality	n	Follow-up time, median (IQR), years	Person-years	CRC cases	Incidence rate per 100 000 person-years (95 %CI)
Pooled screening *	FS/gFOBT	2291	11.8 (10.3 – 16.1)	27636	37	134 (97 – 185)
KPCP	FS	850	10.9 (8.9 – 11.5)	8220	7	85 (41 – 179)
UKFSST	FS	952	16.4 (15.1 – 17.0)	14134	21	149 (97 – 228)
ECP	gFOBT	489	11.6 (9.3 – 13.0)	5282	9	170 (89 – 327)

CI, confidence interval; CRC, colorectal cancer; ECP, English CRC screening pilot; FS, flexible sigmoidoscopy; gFOBT, guaiac fecal occult blood test; IQR, interquartile range; KPCP, Kaiser Permanente CRC prevention program; UKFSST, UK Flexible Sigmoidoscopy Screening Trial.

* KPCP, UKFSST, and ECP pooled data.

With higher risk defined according to the IA study risk factors 14 15 , 45 % of screening participants were classified as higher risk. Of the individual screening cohorts, the ECP had the greatest proportion of higher-risk participants (54 %) ( **Table 2 s** ). Consistent with this, the ECP had the greatest proportion of participants with adenomas ≥ 20 mm or with high-grade dysplasia ( Table 1 ). In comparison, among BCSP participants for whom we had data, 66 % were classified as higher risk ( **Table 2 s** ).

Among all screening participants, the CRC incidence rate after baseline was twice as high in the higher-risk subgroup as in the lower-risk subgroup (184 vs. 92 per 100 000 person-years) ( Table 3 ). The HR for the comparison of CRC incidence rates in the two subgroups was 2.08 (95 %CI 1.07 – 4.06), adjusting for number of surveillance visits (data not shown).

**Table 3:** Unadjusted effect of surveillance on colorectal cancer incidence rates in lower- and higher-risk subgroups.

No. of surveillance visits 1	n (%)	Person-years	CRC cases	Incidence rate per 100 000 person-years (95 %CI)	Effect of surveillance	
					Univariate HR (95 %CI) 2	*P* value 3
Whole intermediate-risk group						
0	499 (21.8)	11146	18	161 (102 – 256)	1	0.01
≥ 1	1792 (78.2)	16490	19	115 (73 – 181)	0.39 (0.19 – 0.81)	
Total	2291 (100)	27636	37	134 (97 – 185)		
Lower-risk subgroup 4						
0	287 (22.8)	6526	7	107 (51 – 225)	1	0.16
≥ 1	971 (77.2)	8630	7	81 (39 – 170)	0.41 (0.12 – 1.38)	
Total	1258 (54.9)	15156	14	92 (55 – 156)		
Higher-risk subgroup 4						
0	212 (20.5)	4620	11	238 (132 – 430)	1	0.03
≥ 1	821 (79.5)	7860	12	153 (87 – 269)	0.35 (0.14 – 0.86)	
Total	1033 (45.1)	12480	23	184 (122 – 277)		

CI, confidence interval; CRC, coloretal cancer; HR, hazard ratio.

1 Number of surveillance visits was included as a time-varying covariate.

2 The univariate HRs were for the comparison of CRC incidence rates in the presence of one or more surveillance visits vs. in the absence of surveillance.

3 *P* values were calculated with the likelihood ratio test.

4 The higher-risk subgroup included individuals who, at baseline, had an incomplete colonoscopy, colonoscopy of unknown completeness, poor bowel preparation, adenoma ≥ 20 mm or with high-grade dysplasia, or proximal polyps. Individuals without any of these baseline characteristics were classified into the lower-risk subgroup.

In the higher-risk subgroup, the CRC incidence rate was lower in the presence of one or more surveillance visits than in the absence of surveillance (univariate HR 0.35, 95 %CI 0.14 – 0.86). In the lower-risk subgroup, the corresponding HR was similar to that for the higher-risk subgroup; however, the estimate was imprecise with a wide 95 %CI as there were only 14 CRC cases (univariate HR 0.41, 95 %CI 0.12 – 1.38) ( Table 3 ).

**Fig. 2:**
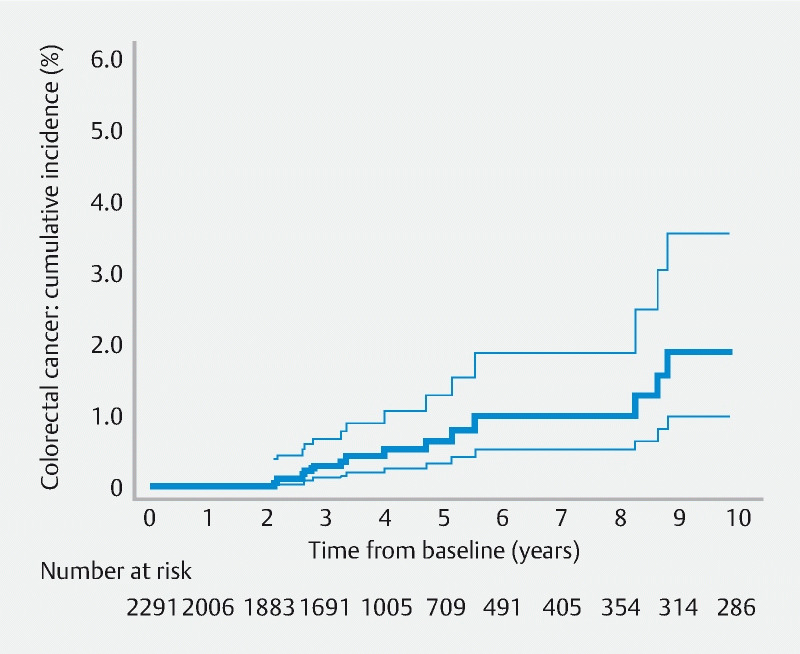
Cumulative colorectal cancer (CRC) incidence after baseline in the absence of surveillance in the screening dataset. The 95 % confidence intervals are shown around the curve. No CRCs were diagnosed in the first 2 years after baseline; five CRCs were diagnosed in years 2 – 3; three CRCs were diagnosed in years 3 – 4; one CRC was diagnosed in years 4 – 5; two CRCs were diagnosed in years 5 – 6; no CRCs were diagnosed in years 6 – 8; three CRCs were diagnosed in years 8 – 9; and no CRCs were diagnosed in years 9 – 10.

Without surveillance, cumulative CRC incidence at 10 years was 1.9 % (95 %CI 1.0 – 3.5) in the whole intermediate-risk group ( Table 4 ; Fig. 2 ), 1.0 % (95 %CI 0.4 – 2.4) in the lower-risk subgroup, and 3.1 % (95 %CI 1.3 – 7.1) in the higher-risk subgroup ( Table 4 ). With one or more surveillance visits, figures were 1.3 % (95 %CI 0.8 – 2.3) in the whole intermediate-risk group, 1.1 % (95 %CI 0.5 – 2.5) in the lower-risk subgroup, and 1.6 % (95 %CI 0.8 – 3.1) in the higher-risk subgroup ( Table 4 ).

**Table 4:** Cumulative colorectal cancer incidence at 10 years in lower- and higher-risk subgroups.

	n (%)	Person-years	CRC cases	Incidence rate per 100000 person-years (95 %CI)	At 10 years’ follow-up		*P* value 1
					CRC cases	Cumulative incidence (95 %CI), %	
Without surveillance (after baseline, censored at first surveillance)							
Whole intermediate-risk group	2291 (100)	11146	18	161 (102 – 256)	14	1.9 (1.0 – 3.5)	
Lower-risk subgroup 2	1258 (54.9)	6526	7	107 (51 – 225)	6	1.0 (0.4 – 2.4)	0.08
Higher-risk subgroup 2	1033 (45.1)	4620	11	238 (132 – 430)	8	3.1 (1.3 – 7.1)	
With one or more surveillance visits (after first surveillance, censored at end of follow-up)							
Whole intermediate-risk group	1792 (100)	16490	19	115 (73 – 181)	15	1.3 (0.8 – 2.3)	
Lower-risk subgroup 2	971 (54.2)	8630	7	81 (39 – 170)	6	1.1 (0.5 – 2.5)	0.23
Higher-risk subgroup 2	821 (45.8)	7860	12	153 (87 – 269)	9	1.6 (0.8 – 3.1)	

CI, confidence interval; CRC, colorectal cancer.

1 *P* values were calculated with the log-rank test to compare cumulative incidence curves in the lower- and higher-risk subgroups.

2 The higher-risk subgroup included individuals who, at baseline, had an incomplete colonoscopy, colonoscopy of unknown completeness, poor bowel preparation, adenoma ≥ 20 mm or with high-grade dysplasia, or proximal polyps. Individuals without any of these baseline characteristics were classified into the lower-risk subgroup.

In the hospital dataset, the median age was 64 years (IQR 59 – 69) and 3360 patients (41 %) were female. Poor bowel preparation and baseline colonoscopies that were incomplete or of unknown completeness were more common among hospital patients than screening participants ( **Table 3 s** ). This was true even with the exclusion of KPCP participants, all of whom were assumed to have had a complete baseline colonoscopy with at least satisfactory bowel preparation (data not shown). A greater proportion of hospital patients had an adenoma ≥ 20 mm, with high-grade dysplasia or villous histology, or proximal polyps at baseline compared with screening participants ( **Table 3 s** ).

During a median follow-up of 8.3 years (IQR 6.0 – 11.6), 140 CRCs were diagnosed among intermediate-risk patients in the hospital dataset, giving an incidence rate of 194 per 100 000 person-years (95 %CI 164 – 229). The proportion of hospital patients classified as higher risk was 73 % ( **Table 4 s** ). The incidence rate of CRC was twice as high in the higher-risk subgroup as in the lower-risk subgroup, adjusting for number of surveillance visits (HR 2.32, 95 %CI 1.43 – 3.77) (data not shown). In the higher-risk subgroup, the CRC incidence rate was lower in the presence of one or more surveillance visits than in the absence of surveillance (univariate HR 0.47, 95 %CI 0.32 – 0.71). The effect of surveillance on the CRC incidence rate in the lower-risk subgroup was unclear as there were few CRCs and the HR estimate was imprecise (univariate HR 0.56, 95 %CI 0.20 – 1.56) ( **Table 4 s** ). Cumulative CRC incidence at 10 years was higher in the higher-risk subgroup than in the lower-risk subgroup ( **Table 5 s** ). These findings mirror those from the screening dataset.

In sensitivity analysis, adenomas with villous histology were associated with an increased CRC incidence rate in the screening dataset ( **Table 6 s** ). When we additionally included villous histology in the higher-risk classification criteria, 47 % of participants were classified as higher risk, which was similar to the proportion in the main analysis (45 %). This is because most participants with villous adenomas (72 %, 105/146) were already classified as higher risk due to other factors (data not shown). As in the main analysis, the CRC incidence rate was twice as high in the higher-risk subgroup as in the lower-risk subgroup when villous histology was included in the classification of higher-risk (data not shown).

## Discussion

This study corroborates our previous IA study, which showed that individuals deemed at intermediate risk following adenoma removal are a heterogeneous group, with differing CRC risk and surveillance requirements 14 15 . While the IA study examined patients classified as intermediate risk following referral to hospital for colonoscopy, the current study showed that these findings also apply in individuals classified as intermediate risk following CRC screening.

For this validation study, we created a screening dataset by pooling data from three screening cohorts on intermediate-risk participants. Classifying the participants into risk subgroups using the IA study baseline CRC risk factors (incomplete colonoscopies, colonoscopies of unknown completeness, poor bowel preparation, adenomas ≥ 20 mm or with high-grade dysplasia, proximal polyps), we found that the incidence rate of CRC following adenoma removal was twice as high in the higher-risk subgroup as in the lower-risk subgroup. This is consistent with what we observed in the IA study 14 15 and when we analyzed a subset of the IA study cohort who were comparable in age to the screening participants (those aged 50 – 74 years). The risk classification criteria therefore appear to universally discriminate two risk subgroups within intermediate-risk individuals, regardless of whether they are from a screening or hospital setting.

The proportion of intermediate-risk individuals classified as higher risk was smaller in the screening dataset than in the IA study (and age-restricted subset of the IA study cohort). This is not surprising as we expect fewer individuals to have higher-risk characteristics in asymptomatic screening populations than in a population of patients referred to hospital. We found that adenomas ≥ 20 mm, adenomas with high-grade dysplasia, and proximal polyps were all less common among screening participants than hospital patients.

Comparing the individual screening cohorts, the ECP had a greater proportion of higher-risk participants than the UKFSST and KPCP, whereas the BCSP had the greatest proportion of all. The gFOBT was used in the ECP and the BCSP cohorts during the period for which we have data. It is possible that individuals undergoing colonoscopy following a positive gFOBT are more likely to have higher-risk findings than individuals undergoing colonoscopy following flexible sigmoidoscopy. Indeed, we found that adenomas ≥ 20 mm and adenomas with high-grade dysplasia were more common among ECP than UKFSST and KPCP participants (who were screened with flexible sigmoidoscopy).

Villous histology was identified as a CRC risk factor in the screening dataset, whereas it was not in the IA study. We showed in a sensitivity analysis that additional inclusion of villous histology in the classification of higher risk did not alter the ratio of higher-risk to lower-risk participants, or the discrimination between the subgroups in terms of CRC incidence rates.

Among screening participants classified as lower risk, CRC incidence rates following adenoma removal were approximately 100 per 100 000 person years in the absence of surveillance. We showed in the IA study that this rate is lower than in the general population, although the screening and IA study cohorts did differ in terms of age, attendance at surveillance, and follow-up time 14 . In a resource-limited setting, and considering the risks of colonoscopy, surveillance should be directed to individuals remaining at increased CRC risk after adenoma removal, compared with the general population. Therefore, as we previously suggested, surveillance may not be warranted for the lower-risk subgroup 14 ; however, as our estimates for this subgroup had wide 95 %CIs owing to few CRC cases, the validity of this assertion remains unclear.

The benefit of surveillance for the higher-risk subgroup of intermediate-risk individuals was clearly demonstrated in the IA study 14 15 . We have now validated this finding in screening participants, showing that surveillance was associated with a significantly reduced CRC incidence rate among higher-risk participants. For lower-risk participants, the effect of surveillance on the CRC incidence rate was unclear as the 95 %CI was wide.

Baseline colonoscopies were of higher quality, with better bowel preparation and higher completion rates, among screening participants than hospital patients. A study comparing the quality of colonoscopies performed by one endoscopist either as part of the BCSP or for non-screening indications similarly found that completion rates were higher in screening than in nonscreening patients 25 . The authors suggested reasons for this, including differences in the age and sex of screening and nonscreening patients, and increased motivation among endoscopists in a screening setting through monitoring of screening performance indicators. A comparison of screening and hospital patients in our analysis revealed that hospital patients were older and a greater proportion were women, in whom colonoscopies are technically more difficult 26 .

Limitations of this study include the low number of CRC cases in the screening dataset, which meant that our estimates lacked precision. Notably, when we stratified participants into lower-risk and higher-risk subgroups, there were only 14 CRCs in the lower-risk subgroup. This prevented clear conclusions from being drawn about the need for and benefit of surveillance among lower-risk individuals. Additionally, we were unable to interpret the relative effects of one vs. two or more surveillance visits on CRC incidence rates.

A further limitation is that our data on baseline colonoscopies were from the mid-1990 s to early 2000 s, whereas data on surveillance colonoscopies were obtained through 2006 for KPCP and 2012 for UKFSST and ECP. As colonoscopy quality has significantly improved over the past two decades 27 , our results may be overestimating CRC risk after baseline colonoscopy and/or underestimating the benefit of surveillance. It is possible that we missed some surveillance examinations, despite endeavoring to obtain complete data. Data were missing on examination quality for KPCP participants so, based on published data indicating that 97 % – 99 % of colonoscopies in the USA are deemed to be complete 21 , we assumed that all KPCP participants had a complete baseline colonoscopy with at least satisfactory bowel preparation. Finally, pooling data from international screening programs employing different screening modalities masked individual variability and limits the generalizability of our findings.

Strengths include the high quality and long follow-up period of the three screening datasets with participants from the UK and USA. Each dataset contained detailed baseline characteristic data, linked endoscopy and pathology data, and follow-up data on CRC diagnoses and deaths. Very few data were missing. By pooling the datasets, we were able to study CRC incidence among more than 2000 intermediate-risk individuals who were followed-up for a median of 11.8 years.

### Conclusion

This validation study showed that screening participants classified as intermediate risk at baseline colonoscopy comprise two risk subgroups, as previously demonstrated for hospital patients. Higher-risk individuals (those with an incomplete colonoscopy, colonoscopy of unknown completeness, poor bowel preparation, adenoma ≥ 20 mm or with high-grade dysplasia, or proximal polyps at baseline) are likely to benefit significantly from surveillance. Among individuals without these characteristics, CRC incidence rates are low following adenoma removal and it is unclear whether surveillance is required. Additional studies with large sample sizes are needed to determine whether lower-risk individuals could safely forego surveillance.

## Supplementary material

Supplementary material Zusatzmaterial
