# Supplementary material for: Heterogeneity in colorectal cancer incidence among people recommended 3-yearly surveillance post-polypectomy: a validation study
Source: Endoscopy. 2020 Aug 19;53(4):402–10. doi: 10.1055/a-1217-0155 (PMC8007389; doi:10.1055/a-1217-0155)
Supplement: Supplementary file 1 — Supplementary material [file 18431supmat_10-1055-a-1217-0155.pdf]

Supplementary material

Ethics approval

For the IA study, ethics approval was granted by the Royal Free Research Ethics Committee (REC) (reference 06/Q0501/45). Approval to process patient identifiable information without consent was granted by the Patient Information Advisory Group (PIAG) under Section 60 of the Health and Social Care Act 2001 (re-enacted by Section 251 of the NHS Act 2006) (reference PIAG 1–05[e]/2006). The use of data from the UKFSST was approved by Public Health England (PHE) Office for Data Release (ODR) (reference ODR1516_379/B). The use of data from the KPCP was approved by the Kaiser Permanente Northern California Institutional Review Board. Ethics approval was not sought by the researchers conducting the ECP, a decision approved by the UK National Screening Committee and Department of Health [1].

Study registration

Two of the studies included in the present analysis are registered with ISRCTN: number ISRCTN15213649 for the IA study [2,3] and ISRCTN28352761 for the UKFSST [4–6]

References

1 Steele RJC. Results of the first round of a demonstration pilot of screening for colorectal cancer in the United Kingdom. BMJ 2004; 329: 133–5

2 Atkin W, Wooldrage K, Brenner A et al. Adenoma surveillance and colorectal cancer incidence: a retrospective, multicentre, cohort study. Lancet Oncol 2017; 18: 823–34

3 Atkin W, Brenner A, Martin J et al. The clinical effectiveness of different surveillance strategies to prevent colorectal cancer in people with intermediate-grade colorectal adenomas: a retrospective cohort analysis, and psychological and economic evaluations. Health Technol Assess 2017; 21: 1–536

4 Atkin W, Wooldrage K, Parkin DM et al. Long term effects of once-only flexible sigmoidoscopy screening after 17 years of follow-up: the UK Flexible Sigmoidoscopy Screening randomised controlled trial. Lancet 2017; 389: 1299–311

5 Atkin WS, Edwards R, Kralj-Hans I et al. Once-only flexible sigmoidoscopy screening in prevention of colorectal cancer: a multicentre randomised controlled trial. Lancet 2010; 375: 1624–33

6 Atkin WS, Cook CF, Cuzick J et al. Single flexible sigmoidoscopy screening to prevent colorectal cancer: baseline findings of a UK multicentre randomised trial. Lancet 2002; 359: 1291–300

Supplementary material

Table 1s. Screening dataset: Baseline patient, procedural, and polyp characteristics in participants with and without surveillance visits.

	Participants with no surveillance visits (n = 499)	Participants with one or more surveillance visits (n = 1792)	*P* value[1]
Sex, n (%)			
Female	154 (30.9)	584 (32.6)	
Male	345 (69.1)	1208 (67.4)	0.47
Age at baseline colonoscopy, years, n (%)			
50-54	56 (11.2)	176 (9.8)	
55-59	121 (24.2)	548 (30.6)	
60-64	167 (33.5)	688 (38.4)	<0.001
65-69	101 (20.2)	309 (17.2)	
70-74	54 (10.8)	71 (4.0)	
Colonoscopy completeness, n (%)			
Complete	475 (95.2)[2]	1725 (96.3)[2]	0.28
Incomplete/unknown	24 (4.8)	67 (3.7)	
Bowel preparation quality, n (%)			
Excellent/good/satisfactory/unknown	490 (98.2)[2]	1752 (97.8)[2]	0.56
Poor	9 (1.8)	40 (2.2)	
Adenoma size, mm, n (%)			
<10	70 (14.0)	195 (10.9)	
10-19	344 (68.9)	1229 (68.6)	0.06
≥20	85 (17.0)	368 (20.5)	
Adenoma histology, n (%)			
Tubular	285 (57.1)	824 (46.0)	
Tubulovillous	175 (35.1)	828 (46.2)	<0.001
Villous	32 (6.4)	114 (6.4)	
Unknown	7 (1.4)	26 (1.5)	
Adenoma dysplasia, n (%)			
Low grade	459 (92.0)	1557 (86.9)	
High grade	35 (7.0)	219 (12.2)	0.005
Unknown	5 (1.0)	16 (0.9)	
Proximal polyps, n (%)			
No	391 (78.4)	1443 (80.5)	0.28
Yes	108 (21.6)	349 (19.5)	

[1] *P* values were calculated with the χ^2 test to compare participants with and without surveillance in the screening dataset.
[2] Data on examination quality were missing for KPCP (Kaiser Permanente CRC prevention program) participants; we therefore assumed that all KPCP participants had a complete colonoscopy with at least satisfactory bowel preparation at baseline.

Supplementary material

Table 2s Proportion of individuals in the screening dataset, individual screening cohorts, hospital dataset, and English Bowel Cancer Screening Programme classified into lower- and higher-risk subgroups.

Dataset	Examination/ screening modality	Whole cohort n	Lower-risk subgroup[1] n (%)	Higher-risk subgroup[1] n (%)
Pooled screening[2]	FS/gFOBT	2291	1258 (54.9)	1033 (45.1)
KPCP	FS	850	530 (62.4)	320 (37.6)
UKFSST	FS	952	505 (53.0)	447 (47.0)
ECP	gFOBT	489	223 (45.6)	266 (54.4)
Hospital	Colonoscopy	8109	2164 (26.7)	5945 (73.3)
BCSP	gFOBT	15,656	5287 (33.8)	10,369 (66.2)

ECP, English CRC screening pilot; BCSP, Bowel Cancer Screening Programme; FS, flexible sigmoidoscopy; gFOBT, guaiac faecal occult blood test; KPCP, Kaiser Permanente CRC prevention programme; UKFSST, UK Flexible Sigmoidoscopy Screening Trial.
[1]The higher-risk subgroup included individuals who, at baseline, had an incomplete colonoscopy, colonoscopy of unknown completeness, poor bowel preparation, adenoma ≥20mm or with high-grade dysplasia, or proximal polyps. Individuals without any of these baseline characteristics were classified into the lower-risk subgroup.
[2]KPCP, UKFSST, and ECP pooled data.

Supplementary material

Table 3s Hospital dataset: Baseline patient, procedural and polyp characteristics.

	**Hospital dataset (n = 8109)**
No. of surveillance visits, n (%)	
0	2853 (35.2)
1	2563 (31.6)
≥2	2693 (33.2)
Sex, n (%)	
Female	3360 (41.4)
Male	4749 (58.6)
Age at baseline colonoscopy, years, n (%)	
50-54	973 (12.0)
55-59	1321 (16.3)
60-64	1858 (22.9)
65-69	2171 (26.8)
70-74	1786 (22.0)
Year of baseline colonoscopy, n (%)	
1984-94	298 (3.7)
1995-9	1008 (12.4)
2000-4	2771 (34.2)
2005-10	4032 (49.7)
Colonoscopy completeness, n (%)	
Complete	6181 (76.2)
Incomplete/unknown	1928 (23.8)
Bowel preparation quality, n (%)	
Excellent/good/satisfactory/unknown	7711 (95.1)
Poor	398 (4.9)
Adenoma size, mm, n (%)	
<10	744 (9.2)
10-19	4741 (58.5)
≥20	2624 (32.4)
Adenoma histology, n (%)	
Tubular	3299 (40.7)
Tubulovillous	3734 (46.0)
Villous	733 (9.0)
Unknown	343 (4.2)
Adenoma dysplasia, n (%)	
Low grade	6450 (79.5)
High grade	1316 (16.2)
Unknown	343 (4.2)
Proximal polyps, n (%)	
No	5581 (68.8)
Yes	2528 (31.2)

Supplementary material

Table 4s Hospital dataset: Unadjusted effect of surveillance on colorectal cancer incidence rates in lower- and higher-risk subgroups.

No. of surveillance visits[1]	n (%)	Person-years	CRC cases	Incidence rate per 100,000 person-years (95%CI)	Effect of surveillance	
					Univariate HR (95%CI)[2]	*P* value[3]
Whole intermediate-risk group						
0	2853 (35.2)	34,685	73	210 (167-265)	1	<0.001
≥1	5256 (64.8)	37,607	67	178 (140-226)	0.51 (0.35-0.75)	
Total	8109 (100)	72,292	140	194 (164-229)		
Lower-risk subgroup[4]						
0	891 (41.2)	10,344	11	106 (59-192)	1	0.27
≥1	1273 (58.8)	8372	8	96 (48-191)	0.56 (0.20-1.56)	
Total	2164 (26.7)	18,716	19	102 (65-159)		
Higher-risk subgroup[4]						
0	1962 (33.0)	24,341	62	255 (199-327)	1	<0.001
≥1	3983 (67.0)	29,235	59	202 (156-260)	0.47 (0.32-0.71)	
Total	5945 (73.3)	53,577	121	226 (189-270)		

CI, confidence interval; CRC, colorectal cancer; HR, hazard ratio.

[1]Number of surveillance visits was included as a time-varying covariate.

[2]The univariate HRs were for the comparison of CRC incidence rates in the presence of one or more surveillance visits vs. in the absence of surveillance.

[3]*P* values were calculated with the likelihood ratio test.

[4]The higher-risk subgroup included individuals who, at baseline, had an incomplete colonoscopy, colonoscopy of unknown completeness, poor bowel preparation, adenoma ≥20mm or with high-grade dysplasia, or proximal polyps. Individuals without any of these baseline characteristics were classified into the lower-risk subgroup.

Supplementary material

Table 5s Hospital dataset: Cumulative colorectal cancer incidence at 10 years in lower- and higher-risk subgroups.

	n (%)	Person-years	CRC cases	Incidence rate per 100,000 person-years (95%CI)	At 10 years' follow-up		*P* value[1]
					CRC cases	Cumulative incidence (95%CI), %	
**Without surveillance (after baseline, censored at first surveillance)**							
Whole intermediate-risk group	8109 (100)	34,685	73	210 (167-265)	68	2.5% (1.8-3.3)	
Lower-risk subgroup[2]	2164 (26.7)	10,344	11	106 (59-192)	10	1.3% (0.5-3.0)	0.004
Higher-risk subgroup[2]	5945 (73.3)	24,341	62	255 (199-327)	58	3.0% (2.2-4.1)	
**With one or more surveillance visits (after first surveillance, censored at end of follow-up)**							
Whole intermediate-risk group	5256 (100)	37,607	67	178 (140-226)	51	1.8% (1.3-2.4)	
Lower-risk subgroup[2]	1273 (24.2)	8372	8	96 (48-191)	6	0.8% (0.3-1.8)	0.06
Higher-risk subgroup[2]	3983 (75.8)	29,235	59	202 (156-260)	45	2.0% (1.5-2.8)	

CI, confidence interval; CRC, colorectal cancer.

[1] *P* values were calculated with the log-rank test to compare cumulative incidence curves in the lower- and higher-risk subgroups.

[2] The higher-risk subgroup included individuals who, at baseline, had an incomplete colonoscopy, colonoscopy of unknown completeness, poor bowel preparation, adenoma ≥20mm or with high-grade dysplasia, or proximal polyps. Individuals without any of these baseline characteristics were classified into the lower-risk subgroup.

Supplementary material

Screening dataset: Long-term incidence rates of colorectal cancer after baseline colonoscopy by baseline risk factors and number of surveillance

	n (%)	Person-years	CRC cases	Incidence rate per 100,000 person-years (95%CI)	Univariate HR (95% CI)[1]	*P* value[2]	Multivariate adjusted HR (95%CI)[3]	*P* value[2]
	2291 (100)	27,636	37	134 (97-185)				
veillance visits[4]								
	499 (21.8)	11,146	18	161 (102-256)	1		n/a	
	794 (34.7)	9394	8	85 (43-170)	0.35 (0.15-0.84)	0.04		
	998 (43.6)	7096	11	155 (86-280)	0.43 (0.18-1.05)			
	738 (32.2)	9185	14	152 (90-257)	1	0.58	1	0.61
	1553 (67.8)	18,450	23	125 (83-188)	0.83 (0.42-1.61)		0.84 (0.43-1.64)	
seline colonoscopy, years								
	232 (10.1)	2391	1	42 (6-297)	1		1	
	669 (29.2)	8816	7	79 (38-167)	1.51 (0.18-12.42)		1.69 (0.20-13.91)	
	855 (37.3)	10,989	18	164 (103-260)	3.13 (0.41-23.71)	0.11	3.45 (0.45-26.22)	0.13
	410 (17.9)	4304	9	209 (109-402)	4.73 (0.60-37.35)		4.99 (0.63-39.46)	
	125 (5.5)	1136	2	176 (44-704)	4.63 (0.42-51.22)		4.13 (0.37-45.74)	
ppy completeness								
e	2200 (96.0)	26,374	35	133 (95-185)	1	0.93	1	0.87
ete/unknown	91 (4.0)	1262	2	159 (40-634)	1.07 (0.26-4.46)		1.13 (0.27-4.71)	
eparation quality								
t/good/satisfactory/unknown	2242 (97.9)	27,004	35	130 (93-181)	1	0.31	1	0.30
	49 (2.1)	632	2	317 (79-1266)	2.31 (0.56-9.63)		2.35 (0.56-9.79)	
size, mm								
	265 (11.6)	3006	3	100 (32-309)	1	0.44	1	
	1573 (68.7)	18,981	23	121 (81-182)	1.16 (0.35-3.88)		1.18 (0.35-3.93)	0.34
	453 (19.8)	5648	11	195 (108-352)	1.83 (0.51-6.55)		1.99 (0.55-7.19)	
histology								
	1109 (48.4)	13,129	14	107 (63-180)	1	0.04	1	0.03
llous	1003 (43.8)	12,250	16	131 (80-213)	1.20 (0.58-2.46)		1.29 (0.63-2.66)	
	146 (6.4)	1791	7	391 (186-820)	3.56 (1.44-8.82)		3.80 (1.53-9.44)	
n	33 (1.4)	466	0	0	n/a		n/a	
dysplasia								
de	2016 (88.0)	24,086	29	120 (84-173)	1	0.11	1	0.07
de	254 (11.1)	3241	8	247 (123-494)	1.97 (0.90-4.32)		2.22 (1.01-4.91)	

Supplementary material

'n	21 (0.9)	309	0	0	n/a		n/a	
polyps								
	1834 (80.1)	22,482	29	129 (90-186)	1	0.55	1	0.59
	457 (19.9)	5154	8	155 (78-310)	1.27 (0.58-2.79)		1.24 (0.57-2.73)	

nce interval; CRC, colorectal cancer; HR, hazard ratio.
: HRs and 95% CIs were estimated using Cox proportional hazards models. Due to the lack of events in participants with unknown adenoma histology, these participants were excluded nating the HRs for adenoma histology. Similarly, participants with unknown adenoma dysplasia were excluded when estimating the HRs for adenoma dysplasia.
ıere calculated with the likelihood ratio test.
ıte adjusted HRs are adjusted for number of surveillance visits. The presented HRs are from fitting a Cox proportional hazards model including the specified variable and number of e visits and the presented *P* values are for inclusion of the specified variable in the model. Due to the lack of events in participants with unknown adenoma histology, these participants ded when estimating the multivariate adjusted HRs for adenoma histology. Similarly, participants with unknown adenoma dysplasia were excluded when estimating the multivariate R for adenoma dysplasia.
veillance visits was included as a time-varying covariate.
